# Global excellence in rheumatology in Latin America: The case of systemic lupus erythematosus

**DOI:** 10.3389/fmed.2022.988191

**Published:** 2023-01-11

**Authors:** Manuel F. Ugarte-Gil, Yurilis Fuentes-Silva, Victor R. Pimentel-Quiroz, Guillermo J. Pons-Estel, Rosana Quintana, Bernardo A. Pons-Estel, Graciela S. Alarcón

**Affiliations:** ^1^Grupo Peruano de Estudio de Enfermedades Autoinmunes Sistémicas, School of Medicine, Universidad Científica del Sur, Lima, Peru; ^2^Department Rheumatology, Hospital Nacional Guillermo Almenara Irigoyen, EsSalud, Lima, Peru; ^3^Department of Medicine, Universidad de Oriente, Ciudad Bolívar, Venezuela; ^4^Grupo Oroño, Centro Regional de Enfermedades Autoinmunes y Reumáticas (GO-CREAR), Rosario, Argentina; ^5^Marnix E. Heersink School of Medicine, The University of Alabama at Birmingham, Birmingham, AL, United States; ^6^Facultad de Medicina, Universidad Peruana Cayetano Heredia, Lima, Peru

**Keywords:** systemic lupus erythematosus (SLE), education, Hispanic, Latin America, South America, research, disparities, prognosis

## Abstract

Systemic lupus erythematosus (SLE) affects more severely non-White populations, due to their genetic background and sociodemographic characteristics. Several studies have evaluated Latin American SLE patients to determine their genetic and clinical characteristics as well as prognostic factors; these studies have not only allowed the development of treatment guidelines aimed at the region but also to support regional and global projects. Additionally, educational activities in Spanish and Portuguese have been started to reduce our patients’ health illiteracy. Despite the relatively low research output from Latin American countries, we consider that studies from our region coupled with the networks developed to increase our capabilities, could be a model for other rare autoimmune diseases.

## Introduction

**FIGURE 1:**
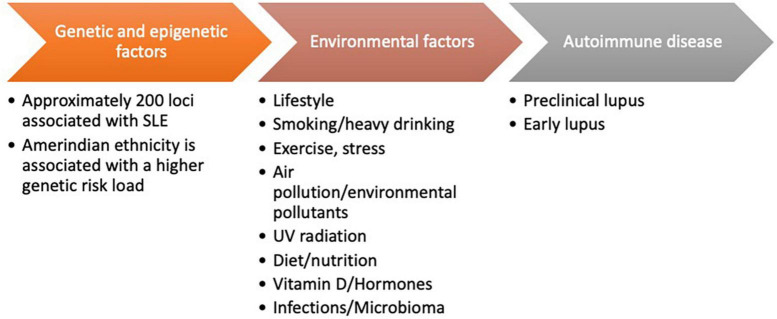
Risk factors of systemic lupus erythematosus (SLE).

Systemic lupus erythematosus (SLE) is an autoimmune disease which affects several organs and is associated with significant morbidity and disability as well as with early mortality. Several factors have been associated with the pathogenesis of SLE; among them, we have, (1): genetic and epigenetic factors comprising approximately 200 risk loci including HLA, signal transducer and activator of transcription 4 (STAT4), interferon regulatory factor 5 (IRF5), B lymphocyte kinase (BLK) family, B cell scaffold protein with ankyrin repeats 1 (BANK1), tumor necrosis factor alpha induced protein 3 (TNFAIP3), tyrosine kinase 2 (TYK2), neutrophil cytosolic factor 1 (NCF1) (1); (2): the racial and ethnic background of the individual; and (3): environmental factors including life style, exercise, stress, smoking, heavy drinking, air pollution, other environmental pollutants, UV radiation, nutritional deficits, vitamin D levels, some hormones, some infections and the microbiome (2, 3). The combination of these factors impacts on the course and outcome of SLE in subjects of different racial and ethnic background (4–7). The risk factors of SLE are shown in Figure 1. In this review we will examine the activities developed to improve the knowledge about SLE in the Latin America (LA) region while at the same time attempting to reduce the inequities observed and to present possible solutions for the future.

## Who are the Latin Americans?

Excluding the USA, Canada and some of the Caribbean countries, the rest of the American continent is called LA, an area inhabited by people with different demographic, biological and cultural characteristics. Excluding Mexico and Central America, the rest of LA is called South America (SA). Regarding SLE, many studies performed in the USA and/or Europe use the term “Hispanic” to refer to patients from Spanish-speaking countries, among them countries from LA. It is necessary, however, to clarify some definitions: Ethnicity refers to shared values, cultural norms, and behaviors of people who are linked by language; such is the case for Hispanics or Latinos, among others; instead, race is defined as a group or identity based on shared phenotypic characteristics including skin color (8). For example, in the LUNAR trial, which was performed in centers from the USA and LA, “races” such as White, Black, Asian/Pacific Islander and Hispanic were considered (9); given this classification, it is very likely that LA patients in this trial were cataloged as Hispanics. It must be noted, however, that the terms race and ethnicity are used in variable form in the literature; in fact, it can be argued that there are no truly distinct racial groups within the human race but rather racial and ethnic groups which share not only phenotypic features but also cultural ones (10). Therefore, considering all previous comments, it can be argued that using the term Hispanic to refer to all LA patients is incorrect as, by definition, it will exclude subjects from the largest LA country who are not Spanish speakers (Brazil) as well as the nearly 45 million people from about 800 indigenous communities living all over the LA region and who speak their own languages.

In addition, as already discussed, Latin Americans are a heterogenous group of people across the region: they have distinctive biological, genetic, and sociocultural features among them. As to their genetic background, they share European, Amerindian (Aztec, Mayan, Quechuan, Aymaran, and other indigenous groups), African and Asian ancestral genes (11). For example, among lupus patients included in the LUMINA (for *Lupus in Minorities: Nature versus Nurture*) cohort, Hispanics from Texas had mostly Amerindian ancestral genes (48%) while those from Puerto Rico have mostly African ones (45%) (12). As to the GLADEL (for *Grupo Latino Americano de Estudio de Lupus*) subjects, they are also a heterogenous group; the majority are Mestizo (of mixed European an Amerindian ancestry) but there also of White, African-LA, pure Amerindian and even of Asian ancestry. In addition, in studies conducted by GLA-GENLES (for *Genoma de Lupus Eritematoso Sistémico*), a consortium developed to evaluate genetic factors in the SLE Latin American population, admixture in LA was higher than in Europe (40.7% American Indian, 49.4% European, 6.2% West African and 3.7% Asian in LA in comparison to 94.6% European, 2.0% American Indian, 1.8% Asian, and 1.6% West African in Europe) (13). Furthermore, admixture can differ among populations within the same region and/or country as has been shown in studies conducted in Argentina and Mexico (14, 15). And in the GALA II (for *Genes-environments and Admixture in Latino Asthmatics*) study, Mexican Americans were found to be mostly of Native American ancestry while Puerto Ricans were found to be mostly of European ancestry (8). Finally, the Latin American Study Group of rheumatic diseases in Indigenous peoples (GLADERPO) has shown that the prevalence of rheumatic diseases varies among these LA communities; for example, the prevalence of rheumatoid arthritis was 0.5% in Raramuris (Mexico) but it was 3.0% in Qom (Argentina) and 3.2 in Whichi (Argentina) (16–18); these data suggest that we need to work harder on the study of genetic ancestry and its association with autoimmune diseases. Given these considerations, it is not correct to call Hispanics to all people from LA, lupus patients included.

The aim of this review is to highlight and emphasize the importance of studies performed in LA populations and how they have contributed to the knowledge of this disease.

## Relevance of the studies performed in Latin America

**FIGURE 2:**
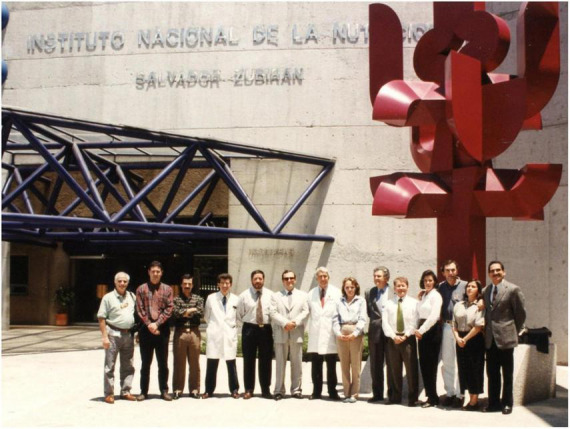
First meeting of the Grupo Latino Americano de Estudio de Lupus (GLADEL) group July 1997. Originally published in https://www.gladel.org/contenidos/2020/09/17/Editorial_2933.php.

Undoubtedly, the most important contributions to a better understanding of SLE in LA have come from the GLADEL group. This network of lupus investigators was started by the late Donato Alarcón-Segovia (from Mexico) and Bernardo A. Pons-Estel (from Argentina) in 1997 and included physicians from the region with expertise in the diagnosis and management of SLE (Figure 2).

The GLADEL group set three main objectives: (1). To establish an inception, multiethnic and multinational lupus cohort in order to obtain data from SLE patients from the region, (2). To develop a LA network of lupus researchers, with the aim of addressing different topics and contemplating the inclusion of senior and junior researchers to assure its continuity over time, and (3). To enhance the understanding of lupus within the LA community, including patients, their relatives, the general population, medical students and primary care physicians, among others.

The GLADEL cohort includes 1,480 patients diagnosed within the previous 2 years in 34 centers from 9 LA countries (19). Most of the patients included (43.6%) are Mestizo and (40.9%) Caucasian, while a smaller proportion is constituted by (11.8%) African (A)-LA (ALA), and (3.7%) by “other” (mainly pure Amerindian and Asian descendant) groups. This distribution is representative of the ethnic composition of LA. In their first 2004 publication, the constitution of this cohort and its general features were described. Socioeconomic status (SES), medical care coverage, and years of formal education were significantly higher among the Caucasian patients. ALA and Mestizo patients were younger at disease onset, had more severe disease with a higher frequency of renal disease and maximum disease activity; they also die at a significantly younger age (19).

GLADEL has shown that understanding the ethnic diversity of our region is essential for approaching patients with lupus, as demonstrated in more than ninety publications, either limited to GLADEL or them being collaborative (20). A relevant contribution of this LA network has been the recognition of the impact of being Mestizo has in renal disease occurrence; it has been observed that these patients have more severe renal involvement and worse outcomes. Furthermore, the Mestizo patients are at an increased risk not only of developing renal involvement overall but of doing so earlier (21–23). The use of antimalarials (AM) has been shown to be beneficial in terms of these patients’ overall survival but also in delaying the occurrence of renal disease and preventing renal failure.

Another GLADEL contribution has been the recognition that Mestizo and ALA ethnicities are predictive of higher disease activity at baseline, while AM use, having medical coverage, and having a longer time to criteria accrual are protective (6). Moreover, GLADEL has shown the association between some clinical manifestations, such as discoid lupus erythematosus being protective of the occurrence of lupus nephritis (LN) while bullous lupus represents an increased risk for it (24, 25).

In relation to the occurrence of renal damage in LN patients, AM use, and a higher SES have been shown to be protective of its occurrence while male gender, hypertension and renal activity have been found to be predictive of it (26). It is worth noting as well, that AM use was protective against serious infections in SLE patients (27).

In LA, many individuals live in rural areas. GLADEL has studied patients living in these areas and found that these patients, in comparison to those living in urban areas, are more frequently Mestizo and younger at SLE diagnosis, have fewer years of formal education, lower SES and less medical coverage. Moreover, these rural patients experience more active disease at diagnosis and renal disease occurrence over time, but no long-term worse outcomes in terms of disease activity, renal damage, overall damage, and mortality (28).

Another focus of this cohort has been the study of factors predisposing to SLE flares and their impact. In this sense, GLADEL has shown that older age at diagnosis and more frequent use of AM are protective, while the use of azathioprine and higher disease activity at baseline are predictive of flares (29). Furthermore, the number of flares, regardless of their severity, increases the risk of damage accrual, regardless of other known risk factors (30). To prevent damage and mortality, the treat to target (T2T) strategy is being implemented worldwide. The aim of this strategy in SLE, is to achieve remission and low disease activity state (LDAS); this has also been studied in GLADEL. It has been demonstrated that the absence of mucocutaneous, renal, and hematologic involvement, the use of immunosuppressive drugs, and lower disease activity early in the course of the disease are predictive of remission and that older age is predictive of LDAS (31). In still another study, being on remission was associated with a lower risk of new and serious damage while being on LDAS was associated with a lower risk of new damage after adjusting for confounders (32).

In 2018, major steps toward improving treatment for LA lupus patients were taken by GLADEL, which with the collaboration of the Pan American League of Associations of Rheumatology (PANLAR), produced the first SLE treatment guidelines for our region. This document resulted from the concerted effort of LA lupus experts (33); special considerations were taken in relation to the impact of racial/ethnic background and SES on lupus outcomes and treatment response. Likewise, cost and availability of specific medications were also taken into consideration since they can affect adherence and are quite relevant in the decision-making process.

Further important contributions have been made by other research groups in LA. Quality of life (QoL) is affected in patients with SLE; several sociodemographic characteristics common in underdeveloped LA countries such as ethnicity, poverty, lower educational level, and inadequate social support have been found to be associated with an impaired health-related (HR) QoL. The Almenara Lupus Cohort from Lima, Perú has contributed important advances to this field; in this cohort, constituted by over 200 SLE patients, severe but not mild-moderate flares were associated with a lower HRQoL, independently of other well-known risk factors for this endpoint (34). Contrarywise, achieving LDAS and remission were associated not only with a better HRQoL in general, but also with improvements in patient’s physical health, pain, planning, burden to others, emotional health, and fatigue scales of the lupus QoL questionnaire (35). Additionally, a Mexican study from the *Universidad de Puebla* has reported the association between a poor HRQoL and the presence of depression, fibromyalgia, disease activity and damage (36).

The epidemiology of SLE has been studied only in certain parts of the region. In a well-designed Argentinean study conducted in subjects from a private health system insurance organization in Buenos Aires, an incidence rate of 6.3 (4.9–7.7) per 100,000 person-years for the total population was found; the sex distribution was of 8.9 for women and 2.6 for men. In terms of the prevalence rates, as of 1 January 2009, there were 58.6 cases of lupus for the total population, 83.2 for women and 23 for men per 100,000 inhabitants (37). Another study performed in Tucumán, one of the 23 provinces that encompasses Argentina, and in which the diagnosis was made only by rheumatologists, lower incidence and prevalence rates than the ones reported for Buenos Aires were found (incidence between 1.8 and 4.2 per 100,000 person-years in 2005 and 2012, respectively; prevalence 34.9 cases/100,000 inhabitants) (38).

Basic research performed at the Universidad de Antioquia, Medellín, Colombia has been of great importance in understanding the role of urinary biomarkers in lupus patients. After studying 120 SLE patients, they found that neutrophil gelatinase associated lipocalin (NGAL), monocyte chemoattractant protein 1 (MCP-1), urinary transferrin and ceruloplasmin were potential biomarkers for LN which may help differentiating active from inactive LN (39, 40). Furthermore, in a Mexican study from the *Hospital General de Mexico Dr. Eduardo Liceaga*, urinary levels of TWEAK (tumor necrosis factor-like weak inducer of apoptosis) were found to be associated with renal activity due to lupus (41). These studies have been the basis for the conceptualization and development of the new GLADEL cohort, named GLADEL 2.0 project.

Finally, the Mexican group from the *Instituto Nacional de Nutrición Salvador Zubirán* has reported an association between the presence of HLA-DR3 and SLE (42) while the *Instituto Nacional de Genómica*, also from Mexico, has reported an association between TLR-7 gene copy number and childhood-onset SLE (43).

Vaccination is of great importance in SLE to prevent infections; important advances in this field have come from Brazilian researchers spearheaded by the group of the *Universidade de Sao Paulo*. Regarding the prevention of the influenza A virus with H1N1/2009 vaccine, these investigators found that the response to this vaccine is diminished in SLE patients receiving immunosuppressive (IS) therapy while the use of AM seems to restore this immunogenicity (44). The yellow fever virus is found in tropical and subtropical SA areas and can cause severe bleeding leading in some cases to a fatal outcome. In a study which included 67 SLE patients out of a total of 159 with autoimmune rheumatic diseases (AIRD) and the same number of controls, 84% of these AIRD patients, despite being treated with low IS doses, reached seroconversion while experiencing neither disease activity flares nor serious adverse events during the 30 days following the vaccination. Finally, the COVID-19 pandemic has caused significant morbidity largely driven by demographic factors, comorbidities and untreated or active SLE (45). Therefore, it has been an important achievement to conduct a clinical trial in Sao Paulo, Brazil where the immunogenicity of an inactivated SARS-CoV-2 vaccine (Sinovac-CoronaVac) and the influence of different medications in SLE as well as their safety, was assessed. These investigators found that the Sinovac-CoronaVac has a moderate immunogenicity in SARS-CoV-2–naive SLE patients with an excellent safety profile. They further demonstrated that hydroxychloroquine may improve seroconversion, whereas prednisone and mycophenolate mofetil (MMF) had a major deleterious effect in vaccine response, reinforcing the need to investigate the role of temporary MMF withdrawal or a vaccine-booster dose (46). Some of the most important SLE studies are depicted in Table 1.

**TABLE 1:** Key findings in systemic lupus erythematosus (SLE) studies from Latin America.

Pathogenesis		References
Genetic	Several genes have been associated with SLE in Hispanic populations; IRF5 has been strongly associated in this group.	(4, 42, 43)
**Biomarkers**		
Lupus nephritis	NGAL, MCP1, TWEAK urinary transferrin and ceruloplasmin have been found to be relevant.	(39–41)
**Disease characteristics**		
Disease activity	Mestizo and African-Latin American racial and ethnic background have been associated with higher disease activity early in the course of the disease while antimalarial use and having medical coverage have been found to be protective.	(6)
Lupus nephritis	Discoid lupus has been found to be protective of its occurrence while bullous lupus has been associated with a higher risk of its occurrence.	(24, 25)
**Outcomes**		
Remission/low disease activity	Lower risk of damage, lower rate of hospitalization, better HRQoL.	(32, 35)
Renal damage	Antimalarial use and higher socioeconomic status have been found to be protective of the occurrence of renal damage while male gender, hypertension and renal activity have been associated with a higher risk of its occurrence.	(26)
HRQoL	Flares have been predictive of poorer HRQoL while depression and fibromyalgia have been associated with poorer HRQoL.	(34, 36)
Vaccination	Immunosuppressive drugs have been associated with a lower response to vaccines like influenza and SARS-CoV2.	(44, 46)

IRF5, Interferon regulatory factor 5; NGAL, neutrophil gelatinase associated lipocalin. MCP-1, monocyte chemoattractant protein 1; TWEAK, tumor necrosis factor-like weak inducer of apoptosis; HRQoL, Health-related quality of life.

## The impact of educational activities

The diagnosis of SLE generates uncertainty in patients and their family members, from the time the diagnosis is made and beyond, as patients will be facing a lifelong disease. SLE treatment guidelines contemplate pharmacological and non-pharmacological treatments to achieve remission and avoid organ damage (33, 47, 48); this implies patients’ prior knowledge about treatment adherence and lifestyle changes leading to improvements in different disease-related outcomes. Many factors such as SES and ethnicity have been linked to adverse outcomes in SLE (49); however, the impact of patient empowerment on these outcomes requires further understanding.

The impact of epidemiological, clinical, therapeutic and genomic studies in SLE has been remarkable in LA (50–52), but we will be remissive if we do not focus on our patients suffering with the disease and in their needs. The SLE patients must become empowered to have better communication with their physicians and thus be able to contribute to improve their prognosis and their QoL, avoid organ damage and increase their life expectancy (53). Low patient activation has been observed in more than one-third of lupus patients, indicating that a large proportion of patients perceive that they are lacking lupus self-management skills (54). Several studies have shown that SLE patients exhibit a poorer QoL when compared to controls (55). In LA, these findings have also been replicated in SLE, showing that a diminished QoL in SLE patients is related to negative disease outcomes (35, 56–61).

Systemic lupus erythematosus is characterized by a chronic remitting course and a wide variety of disease-associated and non-disease-related factors that not only lead to significant health care costs, such as medical consultations and diagnostic and therapeutic procedures, but it also imposes a significant burden on the patients’ HRQoL, work-related disability, and other outcomes that are more difficult to measure (62). To approach educational interventions for these patients, it is important to consider self-management related to health literacy, which refers to the set of knowledge, skills and experiences in health matters that make individuals knowledgeable about their own health status and how to take care of themselves; this in turn, will allow patients to have a correct interaction with physicians, scientific societies and other actors allowing them to contribute their vision and perspectives as patients on equal terms (63).

A clear objective of an educational project aimed at individuals with lupus in LA should be to provide information to patients to empower them to acquire self-management skills. This process may contribute to the social integration of these individuals, to improve their QoL, reduce the possibility of them becoming disabled and to increase their survival.

In order to implement curricular changes and to be able to train physicians with scientific, epidemiological and technological skills that allow them to face social problems as they practice medicine, it is necessary to know the needs of patients (64); however, there are only a few questionnaires that allow understanding the knowledge that patients with SLE have about their disease. The first questionnaire developed for this purpose was the one by Sullivan (65), where various considerations were made about the large number of factors that could influence knowledge; it also highlights that being able to know the gaps in relation to their knowledge about their disease, allows them to intervene in health policies that may help in improving the quality of the care they receive, through better self-management skills. There is a second questionnaire to assess patients’ knowledge of SLE, called “Do you know what I mean?”, developed by Meara et al. (66), validated in Italian by Peta (67) and in Spanish (Argentinean version) by Serrano et al. (68); these validated questionnaires are useful tools as a starting point to determine the patients’ knowledge about lupus, to offer education directed to their needs, as these may be quite variable in LA and even within the same country, due to educational, cultural and economic factors and access to the health care system.

In terms of SLE self-management training programs, the USA has been the pioneer in the Americas with the development of educational programs using the peer model such as the PALS (Peer Approaches to Lupus Self-Management) program (69), and WELL (The Women Empowered to Live with Lupus) (70, 71). While PALS relies on mentoring facilitated by previously trained patients, WELL uses the CDSMP (Chronic Disease Self-Management Program) peer education program established for individuals with chronic diseases. Both programs seek to improve self-efficacy and self-management skills in women of African descent with lupus.

Latin America has undertaken programs aimed at improving knowledge about rheumatic diseases; one of the LA pioneers has been PANLAR beginning with the first patients’ meeting in 2016, which have continued to be held annually since then. In 2018 a new organization, ASOPAN (Pan-American Network of Rheumatic Diseases Patients’ Associations; in Spanish, *Red Panamericana de Asociaciones de Pacientes con Enfermedades Reumáticas*) emerged; ASOPAN brings together a large part of the LA patients associations through which continuous work is being carried out; the first publication from this group is “the patients’ manifesto,” which is the product of the joint work of physicians and patients, where patients’ priority needs, and their perspectives for interventions in health policies in LA are noted. This document clearly aims at empowering an increasing number of patients and closing the gaps in the healthcare system making it a reference for the design of topics for educational projects in the region (72).

Pan-American Network of Rheumatic Diseases Patients’ Associations, in which most of the lupus patient associations in the region participate, also liaises with patient organizations in other countries outside of LA, with the aim of increasing awareness of rheumatic diseases and offering self-management tools to patients with rheumatic diseases. The first experience for training programs for expert patients emerged in the 1,970’s at Stanford University in the USA (73) and in LA: the *Universidad del Paciente y la Familia* (Patient and Family University), a pioneer program in the region working, hand in hand, with patients with rheumatic diseases’ organizations (74). Likewise, PANLAR initiated the expert patient training program in 2020, with the participation of some Latin American countries (ASOPAN); however, all planned activities had to be postponed due to the emergence of the COVID-19 pandemic (72).

A key educational resource in LA is the Let’s Talk About Lupus Program, a comprehensive online program to educate its population about lupus. The first phase of the program was in Spanish, was called *Hablemos de Lupus* and was launched in May 2017 through social media [Facebook (FB), Instagram, Twitter, and YouTube]; these were followed by a dedicated website. A year later, the program was replicated in Portuguese, under the name *Falando de Lupus*, to reach Brazilian audiences. In this program, there are different educational resources: posts (videos, photos), live video-chats that include a brief overview of the topic by experts, followed by public-expert chats and responses to questions/comments posted by the public (75).

Let’s Talk About Lupus users’ satisfaction has been evaluated through an online survey among 952 respondents; 763 (80.1%) agreed and 162 (17.0%) somewhat agreed with the statement that the program helped them to better understand lupus. Users reported that the most helpful resources were the animated videos (46.1%), followed by the live videos with experts (27.3%), the comments posted by the community management team (15.1%), and the peers’ comments (11.5%). In summary, as Drenkard et al commented about this program, the high level of users satisfaction suggests that the program filled a critical information gap (75).

Let’s Talk About Lupus has become a safe virtual community for patients and healthcare professionals, serving as an innovative education model for other rheumatic conditions. However, further evaluation is needed to determine whether users have a more active role in their self-management activities, including communication with their providers and shared decision-making (75).

It has also been useful to conduct quantitative and qualitative patient-centered research to advance our understanding of patients’ beliefs, healthcare needs, and the challenges in managing their disease (76–81). Let’s Talk About Lupus is the largest, and one of the most successful, online programs in Spanish and Portuguese that aims to educate and engage the LA population with lupus.

A barrier to the development of educational programs in LA is the relatively low number of rheumatologists in the region, with one rheumatologist for every 106,838 inhabitants (82), far below what is recommended by the World Health Organization (WHO) and the Spanish Society of Rheumatology (*Sociedad Española de Reumatología, SER*) that have established a standard of one rheumatologist for every 40,000/50,000 inhabitants to ensure proper patient care (83). In addition, clinicians often do not receive formal training to provide self-management tools to their patients.

A recent transnational initiative with the participation of eight Spanish and Portuguese speaking countries, called “Core Curriculum for medical undergraduate students in LA, Portugal and Spain” (LAPS-CCC), aims at increasing awareness and dissemination of educational programs of physician-patient communication in medical schools (84). This initiative aims at redefining health education, to prepare the future physician to integrate patients in self-management strategies, helping them to generate tools that favor a transversal physician-patient relationship that derives in shared decision making that benefits the patients’ health status; this is particularly relevant in diseases such as SLE.

Self-management is especially important for subjects with chronic diseases such as SLE, where only they can take responsibility for their daily care during the course of their disease. For most of these individuals, self-management entails a lifelong task (85). There are many factors involved in this process, depending on patients, physicians and their communication skills, the healthcare system that imposes the dynamics of the consultation and access to the healthcare system, as well as factors such as educational, socioeconomic, family, cultural and societal levels, among others.

When persons with SLE do not receive information or education about their disease, a gap opens up between collective interpretative resources that puts them at an unfair disadvantage when it comes to making sense of their social experiences (86) considering the difficulties derived from having a rare, heterogeneous disease with periods of exacerbations and remissions. It is thus difficult to find support in collective resources due to lack of interpretation and this makes it difficult for those affected to understand their own experiences.

Reconstructing the key elements in the training process of chronically ill persons toward self-management and finally the empowerment of their medical condition, implies giving a resignification to the curriculum in health education, involving dignifying patients with SLE and integrating them into society with equal opportunities, allowing them to assume and resolve the difficulties arising from their health situation, both at a personal level and to know how to navigate the healthcare system and at the societal level by contributing to generate favorable changes in health policies in their favor.

Finally, lupus patient associations in LA are increasingly managing to empower more patients, offer psychological support and advocate for improvements in health policies that favor SLE patients; such is the case of *Agrupación Lupus Chile* with the implementation of the Ricarte Soto law that favors providing financial protection for high-cost diagnosis and medications, lupus included (87). Another example is *Lupus El Salvador* which has established alliances with the Human Rights Ombudsman’s Office, making it possible to protect patients’ rights and improve their access to the health system, especially in conditions of vulnerability (88).

**FIGURE 3:**
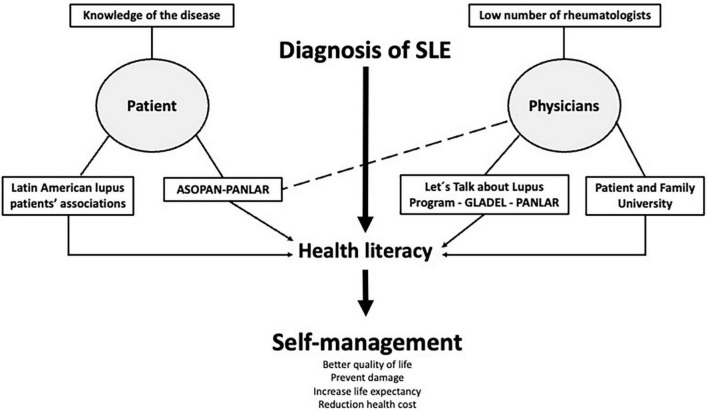
Model of educational activities in Latin America for systemic lupus erythematosus.

In summary, the first step has already been taken, the impact of educational strategies in the region has benefited the SLE patient community, highlighting the importance of having empowered patients who work together with physicians and other health care professionals to achieve success in their treatment, as well as to reduce the overall health costs of this disease. We must review and discuss undergraduate, graduate and postgraduate medical training programs to improve communication skills with patients, promote rheumatology training in the region and improve health policies that favor self-management strategies. The model of educational activities in Latin America is depicted in Figure 3.

## Success and problems

All the data generated from LA and mentioned in this manuscript have been possible even though investigators have faced and still face several problems. These problems influence the possibility of achieving excellence; however, several strategies could be developed.

### Lack of access to healthcare

In LA, and as already noted, most countries have less than one rheumatologist per 85,000 inhabitants; in addition, these rheumatologists are, by and large, located in the largest cities, leaving individuals from rural areas with limited to no access to healthcare (82). As it is not possible to increase the number of rheumatologists in the foreseeable future, it is important to include other specialties, like primary care physicians (P) in the management of these patients. Unfortunately, P do not have enough knowledge about how to diagnose or manage these lupus patients. To solve this problem, GLADEL is launching the program “Is it lupus?”; this program would allow PCPs to improve their knowledge about this disease so it can be recognized promptly and treated appropriately. Additionally, the COVID-19 pandemic forced us to practice telehealth, and this tool could be useful for supporting the management of patients who cannot participate on in-person visits (89).

### Health illiteracy

As SLE is a rare disease, most of the population do not know much about it. Additionally, as it has several and diverse clinical manifestations, the diagnosis is complicated, and, sometimes, patients do not know which specialist they need to visit. To solve this problem, educational projects, like Let’s Talk About Lupus, increase health literacy and improve self-management. Also, as patients get involved in the project, patients could start doing peer-education to other SLE patients (75).

### Lack of information

To define public policy is important to have accurate information about the characteristics of the disease, as well as its possible impact on several outcomes (hospitalization, mortality, costs, among others). The work done by the GLADEL group and others in the region allow us to have information to support the clinical practice guidelines in our region, and this information should also be used to propose public policy about SLE (33).

### Lack of resources and funding

Low-medium income countries (LMICs) do not allocate an adequate amount of money to research (90); however, collaboration (regional and worldwide) could allow us to solve some relevant questions for the region. Some examples of collaboration are GLADEL, which describes the characteristics of LA patients (19); GLA-GENLES, which describes the genetic factors associated with SLE in LA (4); and the COVID-19 Global Rheumatology Alliance, an international project developed at the beginning of the COVID-19 pandemic which has shown the factors associated with poorer outcomes related to COVID-19 infection in SLE patients (45). Additionally, there are some regional grants that support research in the region like the PANLAR Stimulus Award and the H. Ralph Schumacher MD/Journal of Clinical Rheumatology/PANLAR Award, among others (91).

### Inadequate training

**FIGURE 4:**
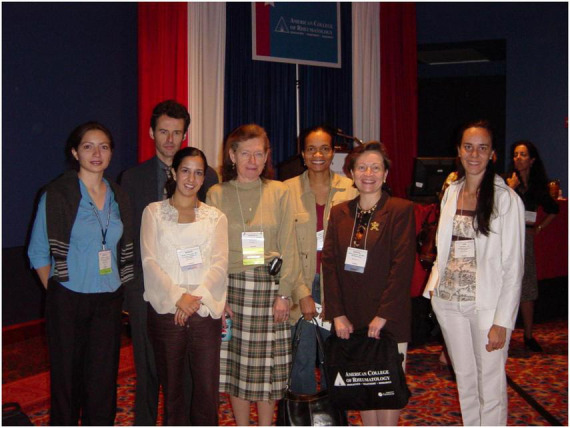
Graciela S. Alarcón with some of the Supporting Training Efforts in Lupus for Latin American Rheumatologists (STELLAR) and/or Lupus in Minorities: Nature versus Nurture (LUMINA) fellows in the mid 2000’s. Published at https://globalrheumpanlar.org/articulo/graciela-s-alarcon-mentora-en-la-esfera-academica-y-en-la-vida-familiar-786.

**FIGURE 5:**
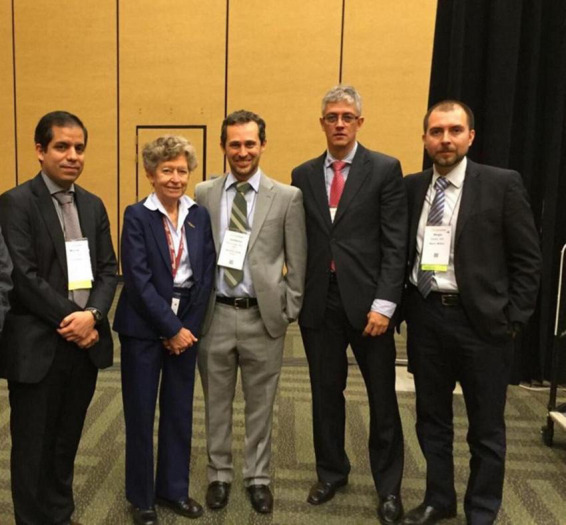
Graciela S. Alarcón with some of the Supporting Training Efforts in Lupus for Latin American Rheumatologists (STELLAR) and/or Lupus in Minorities: Nature versus Nurture (LUMINA) fellows in the mid 2010’s.

As Latin American countries have a relatively low research production, new generations of rheumatologists are not properly trained/training on research. To solve this situation, several strategies have been used, one of them the engagement of local and international experts for the training and mentoring of these young rheumatologists. Collaborative projects allow mentees to identify possible mentors outside their own countries. The LUMINA cohort investigators, with a grant from Rheuminations, Inc. established the STELLAR (for Supporting Training Efforts in Lupus for Latin American Rheumatologists) program which allowed the training of several young rheumatologists from the region which are now conducting research in their own countries, and also mentoring new rheumatologists (Figures 4, 5; 92).

### Lack of dedicated time

In most places in LA, research is not considered part of the job description of practicing physicians, even for those in academic settings. In addition, rheumatologists, and other specialists, usually need to work in several places to reach a desirable income. This situation forces these specialists to spend more time in their private practices than in doing research. However, there have been some improvements in some countries as research has been encouraged and supported (at least partially) by some governments.

## Conclusion

There are several examples of success in LA, particularly, in SLE. We have data that shows not all “Hispanics” are the same. We have been able to characterize the genetics of our lupus population, and we have been able to improve the knowledge about the disease in our patients. This progress has not been achieved without surmounting numerous difficulties; but, collaboration, mentorship as well as the support of some regional organizations have impacted on the capacities of LA rheumatologists, their research production and the information provided to patients. For example, it is worth mentioning that this year marks the 25th anniversary of GLADEL’s inception. These efforts should be encouraged and extrapolated to other diseases in the region and worldwide.

## Author contributions

MU-G, YF-S, VP-Q, GP-E, and RQ wrote the manuscript. GA and BP-E supervised the project. All authors edited and critically revised the manuscript for important intellectual content and gave final approval for the version to be published.
